# Show and tell: disclosure and data sharing in experimental pathology

**DOI:** 10.1242/dmm.026054

**Published:** 2016-06-01

**Authors:** Paul N. Schofield, Jerrold M. Ward, John P. Sundberg

**Affiliations:** 1 Department of Physiology, Development and Neuroscience, University of Cambridge, Cambridge CB2 3EG, UK; 2 The Jackson Laboratory, Bar Harbor, ME 04609, USA; 3 Global VetPathology, Montgomery Village, MD 20886, USA

**Keywords:** Mouse, Histopathology, Data sharing, Peer review, Reproducibility

## Abstract

Reproducibility of data from experimental investigations using animal models is increasingly under scrutiny because of the potentially negative impact of poor reproducibility on the translation of basic research. Histopathology is a key tool in biomedical research, in particular for the phenotyping of animal models to provide insights into the pathobiology of diseases. Failure to disclose and share crucial histopathological experimental details compromises the validity of the review process and reliability of the conclusions. We discuss factors that affect the interpretation and validation of histopathology data in publications and the importance of making these data accessible to promote replicability in research.

## Abstract (precis)

**Summary:** Reproducibility of findings in experiments using model organisms has recently become a source of concern, particularly for translational science. We discuss factors affecting the interpretation and reliability of experimental pathology findings in the mouse, and how disclosure and transparent reporting are crucial for replicability.

## Reproducibility: an age-old quest

*“A good physiological experiment … requires that it should present anywhere, at any time, under identical conditions, the same certain and unequivocal phenomena that can always be confirmed.”* –

Johannes Peter Müller, German physiologist and comparative anatomist (1801-1858).

Illustrated by this quote from Müller, a formal concept of reproducibility existed even in the beginnings of modern experimental biology. Today, the ability to reproduce experimental findings remains essential for the forward movement of science and the application of laboratory findings to the clinic.

There has been much discussion in recent years about the reported irreproducibility of preclinical data obtained using animal models (Begley and Ioannidis, 2015; Collins and Tabak, 2014; Freedman et al., 2015; Mak et al., 2014) and the cost to the success of both translational research and the public purse. The inability to replicate drug-target discovery studies and to reliably replicate phenotype observations from the literature (Begley and Ellis, 2012) has caused profound concern amongst investigators and funding agencies alike, which has been mirrored in discussions and commentaries in the literature. Several issues are tied up inextricably in these discussions. Reproducibility depends first and foremost on the accurate and comprehensive reporting of key experimental procedures and conditions, but it also depends on the open availability of the data, protocols and reagents (Schofield et al., 2009). Both aspects of reproducibility are crucial, and require distinct solutions.

These discussions have stimulated action on the part of funding agencies and scholarly societies to improve the reliability and reproducibility of animal-model-based research. For example, the US National Institutes of Health (NIH) has recently introduced guidelines on rigor and transparency for grant applications^1^, and the Federation of American Societies for Experimental Biology (FASEB) held a meeting in August of 2015 to help members prepare to comply with these new requirements. Scientific journals also have a crucial role in improving reproducibility; acting as effective gatekeepers of knowledge. This depends on enforcement of journal policies on reporting and transparency, and making data sharing and openness part of the community norms that should be expected by reviewers and authors. The ability of researchers to replicate published studies depends on the quality of the peer review process and insistence on full disclosure of data and methods.

Histopathology has been, and remains, a central approach to the characterization of phenotypes of model organisms and humans alike, and has a very important role in both high-throughput and hypothesis-driven characterization of mouse mutants as models for human diseases and therapeutic endpoints (Adissu et al., 2014; Schofield et al., 2012). Replicability of histopathological findings has a major impact on the interpretation of the effects of a mutation or drug treatment, yet the reporting of histopathology in many, if not most, papers often lacks transparency and completeness. Moreover, although scientific errors in pathology can largely be avoided by careful planning and the use of expert, board-certified pathologists, the ability to detect these errors in peer review depends on comprehensive disclosure of the details of animals and protocols. Recommendations on best practice in mouse experimentation were recently discussed in an article from Justice and Dhillon (2016) and, earlier this year, Scudamore and colleagues proposed a set of minimal standards for the reporting of histopathological findings in experimental pathology data (Scudamore et al., 2016). The guidelines cover technical aspects of histopathology comprehensively, such as the use of proper terminology and declaration of the level of skill of the pathologist, but there are additional parameters that affect the type, frequency and severity of lesions, which cannot be assessed without full disclosure from investigators. Of course, histopathology assessed by non-pathologists, or those without specialized training in laboratory mouse pathology, can often lead to lack of reproducibility due to lack of expertise. Surprising though it may seem, basic information, such as strain, mutated gene allele, age and sex of mice, is often lacking or incorrect in study descriptions, and such omissions can have a profound effect on the reproducibility of histopathological findings or even the validity of the interpretation. These issues are compounded by the diverse effects of environment, microbiome, diet and breeding history, as discussed below. As early as 1993, variations in the phenotypes of non-obese diabetic (NOD) mice in different laboratories were investigated using a questionnaire, which demonstrated the impact of factors such as the source of the mice, colony size, breeding, diet, specific-pathogen-free (SPF) status and disease control (Pozzilli et al., 1993). Below, we explore those factors that have been identified as important in the interpretation of phenotypes in mouse experimental pathology, and identify challenges in capturing and reporting them in the quest for enhanced reproducibility.

## Age, sex, strain, and allelic mutation: ASSAM

Mouse genetic nomenclature is a crucial part of the materials and methods of any paper. This includes the strain designation as well as any mutated genes under investigation. Not only should the gene be listed and listed correctly, using the most current name and symbol [refer to Mouse Genome Informatics (http://www.informatics.jax.org/marker) and to Sundberg and Schofield (2009)], but the specific allele as well. For example, although C57BL/6J and C57BL/6NJ are very similar, the latter substrain is homozygous for retinal degeneration 8 (*Crb1^rd^^8^*) (Mattapallil et al., 2012), which must be corrected for ocular studies (Low et al., 2014). Therefore, simply referring to C57BL/6 mice or B6 is inadequate, and without this specification one might interpret background lesions as being due to an experimental manipulation, especially if the background phenotype is partially penetrant or varies in severity from mouse to mouse. If the strain is incorrectly represented, or even omitted, then a reviewer cannot assess the results. Increasing differences between 6N and 6J are now coming to light (Simon et al., 2013) and it is important that investigators use the appropriate substrain as control.

One issue not often discussed, persisting like an elephant in the sitting room, is the existence of ‘passenger’ variants or mutations that remain close to a manipulated target gene during congenic crosses (Vanden Berghe, et al., 2015). Although it is often assumed that ten crosses onto a new background are sufficient to consider the strain stably inbred, recent examples show that this might not always be true, and passenger mutations have recently been found to account for the defective IL-1β production of a *Casp1*-null (*Casp1^tm1Sesh^*) mouse strain that was found to carry a null passenger mutation in the neighboring *Casp11* gene (Kayagaki et al., 2011), the actual cause of the phenotype. Not knowing how many crosses were undertaken onto a new background seriously reduces the ability of reviewers to assess the likelihood of the impact of passenger mutations on the phenotype; therefore, it is crucial that this detail is always reported.

Similarly remarkable is the failure of many investigators to specify the sex and age of their mice. All inbred strains are prone to developing a variety of diseases spontaneously. In many cases the frequency of these diseases increases with age and often has a sex bias (Sundberg et al., 2011, 2016). These predispositions need to be known by investigators in order to differentiate them from changes associated with the mutation under investigation. A recent comprehensive text-mining exercise of more than 15,000 experimental mouse papers (Flórez-Vargas et al., 2016) showed that, although the percentage of papers reporting the sex and age of mice has increased over the past 20 years, only about 50% of the papers published in 2014 reported both of these traits. However, this is marginally worse than when a similar study was conducted in 2009 (Kilkenny et al., 2009). The correct interpretation of histopathological data, like many other types of phenotype data, often depends on knowing both age and sex. Both of these criteria are thus included in the ARRIVE guidelines (Animal Research: Reporting of *In Vivo* Experiments), which were introduced with the aim of improving standards in reporting of biological research (Kilkenny et al., 2010). However, a recent estimate of compliance with the guidelines suggested that there has been little improvement since the guidelines were released in 2010 (Baker et al., 2014). Although many journals include these criteria as a requirement for publication in their instructions for authors, not all editors or reviewers mandate compliance, often in efforts to save space. One challenge in complying with the guidelines, highlighted by Karp et al. (2015), is the availability of standardized language and data structure in which experiments are formally described. The integration of these formal standards into animal data management tools and databases should help with both data capture and encouragement of good practice. Nonetheless, a clear statement by funding agencies and in journal editorial policies, requiring that the ARRIVE guidelines be adhered to, will be a crucial factor in improving the reporting of *in vivo* experiments across the bioscience community; this will need to be matched by the will to enforce the requirements.

## Impact of the microbiome

The reporting of environment, health (pathogen) status and diet is widely accepted to be important in behavioral and metabolic studies (Nature Biotechnology Editorial, 2009; Reardon, 2016), and yet all can have profound effects on the nature and severity of histological lesions in experimental mice (Ward, 1997). The unprecedented ability to analyze and characterize the microbiome using next-generation sequencing (NGS) has brought about a revolution in our appreciation of what used to be ‘known unknowns’ in factors affecting animal phenotypes. We now know that there is a clear genetic and environmental impact on the gut microbiome and that this in turn can influence experimentally induced phenotypes (Campbell et al., 2012), discussed in an excellent review by Laukens et al. (2016). Factors such as housing, local animal house microbiota and diet can all affect the mouse gut microbiome (Nguyen et al., 2015). It was recently shown that even between the C57BL/6N and C57BL/6J substrains, mice have differences in intestinal microbiome (Newberry et al., 2015) and, across a whole range of strains, individuals show much more intrastrain conformity than between strains (Kovacs et al., 2011). Of concern is the observation that mice of the same strain from different vendors have different intestinal flora (Ericsson et al., 2015) and that gut flora have been found to be significantly heritable through the maternal line for several generations, suggesting that, optimally, only related individuals should be used as experimental controls to reduce microbiome variation.

Both the presence of individual pathogens or the specific gut or skin microbiome has increasingly being shown to affect the severity or type of mutant mouse phenotypes (reviewed by Treuting et al., 2012; Barthold, 2004). There are many such examples, often related to inflammatory or immune phenotypes; for example, the presence of *Helicobacter* promotes tumors in the colons of *Apc^Min^* or 129-*Smad3^tm/Par^*/J mice (Newman et al., 2001; Maggio-Price et al., 2006) and the strain-specific colitis phenotype of IL10-deficient mice depends on differences in microbiome composition (Buchler et al., 2012). Remarkably, the spontaneous colitis phenotype in mice lacking *Tbx21*, a regulator of the innate immune response (Garrett et al., 2007), depends on the presence of only 12 microbial species (Powell et al., 2012). There could also be sex-dependent effects of the microbiome; for instance, male-specific differences in microbiome composition have been shown to provide protection from type 1 diabetes in NOD mice (Markle et al., 2013).

As part of reports attributing a specific phenotype to a particular mutation or variant, information about the vendor, stock numbers and dates of acquisition should be recorded, and there should be meticulous documentation of details relating to backcrossing and pedigree. Tied in with what we already know about variations of phenotypes between even closely related substrains (Simon et al., 2013), the additional impact of microbiome and health status needs to be considered carefully, and at the very least relevant details reported to allow a more complete assessment of the experimental findings, especially findings that could have preclinical and translational applications.

## Impact of diet

The importance of diet is illustrated by studies involving mutations that affect calcium metabolism. When mice that model ectopic mineralization are placed on diets with increased phosphate and reduced magnesium (‘acceleration diet’), the speed of development and the distribution of lesions are changed (Li et al., 2014). For example, epicardial mineralization and fibrosis are commonly observed in old KK/HlJ mice on standard mouse diets (Berndt et al., 2014) but, when the mice are placed on the ‘acceleration diet’, mineralization and fibrosis becomes multifocal, involving the entire myocardium and infrequently the epicardium alone (Quiaoli Li, Jouni Uitto and J.P.S., unpublished data).

Much of our knowledge on the impact of diet on phenotypes pertains to the effects of dietary fat on metabolism; however, non-metabolic phenotypes could also be affected. For example, a high-fat diet greatly exacerbates the cardiac development phenotype of *Cited2* loss-of-function mutants (*Cited2^tm1Bha^*) (Bentham et al., 2010). Long-term exposure to diets supplemented with methyl donors, including betaine, methionine and folate, induce wide-ranging heritable and reproducible changes in epigenetic markers. The affected genes include those in pathways controlling gene expression and embryonic development (Li et al., 2011). Moreover, small changes in dietary composition affect placental development and histological remodeling, which have been suggested to influence the intrauterine programming of life expectancy (Coan et al., 2011).

Commercial ‘standard chow’ diets vary substantially depending on the producer. The total fat content can vary between 3.5 and 12%, and the type of fat can also vary, with animal fat or soy oil used in different formulations, together with different levels of vitamin E, other anti-oxidants and phytoestrogens (Reliene and Schiestl, 2006). In a survey on the composition of diets reported as being ‘high fat’, Warden and Fisler (2008) reported that only 5 out of the 35 papers they examined that claimed to use animal models of high-fat feeding had actually compared two diets differing only in the relative amounts of fat and carbohydrate, and 34% had insufficient data on diet to allow the studies to be reproduced.

It is clear, therefore, that, in many cases, replication of experiments could be confounded by a lack of information about the experimental diet used. Diet could affect the pathobiology of mutants directly or indirectly through effects, for example, on the microbiome of the gut as discussed above (Nguyen et al., 2015) or even through changes in behaviour (Pyndt Jorgensen et al., 2014). The possibility that some investigators might not know how their animals are being fed raises serious concerns for interpretation of data as well as its reproducibility.

Above, we have discussed only some of the factors that influence the nature of lesions, particularly inflammatory and neoplastic, that can be detected through histopathology. Although appreciation of the potential confounding nature of some of these environmental factors on interpretation of pathological findings is important, this is not a plea for experimental standardization. It has become clear that heterogeneity of environment might be very important in improving mouse models of human diseases precisely because environmental parameters are not standardized across all animal facilities, mirroring the diversity of environments in which humans live (Richter et al., 2009; Reardon, 2016; Beura et al., 2016). What it does highlight though, is the importance of the capture and presentation of these parameters in publications. As yet, they are not covered in detail by the ARRIVE guidelines and it might now be time to consider increasing the granularity of the guidelines, within the scope of what is reasonable, as balanced against what might significantly affect the interpretation of results.

## An image problem

The primary data behind histopathological findings are almost always photo- or electron micrographs and, as discussed in Scudamore et al. (2016), the space for publishing selected images in papers is often limited or restricted. In some cases, images are not published at all but, more often, small ‘representative’ images are made available. Again, this makes it very difficult to assess the quality of the study and the validity of the interpretation. This size limitation thus presents an obstacle to reader interpretation of published data, but, in addition, the lack of raw photomicrographs in submitted manuscripts makes it an act of faith for a reviewer to make a judgment on a study during the review process. Furthermore, most manuscript reviews of studies using mouse models still do not include assessment by experienced qualified pathologists. Mandated sharing of primary data is now becoming standard for many funding agencies^2^, and deposition of primary data in suitable repositories has become a condition of publication in many journals. However, with the exception of supplementary information sections in journals, there are no dedicated platforms for the sharing of many kinds of data, and most publicly available repositories are for specific types of data, e.g. microarray gene expression datasets. To date, there has been no large-scale commitment of major funding agencies to provide platforms for the provision of experimental pathology data (specifically, whole-slide images).

Pathbase (www.pathbase.net) is an example of a community resource that provides a platform for sharing images for mouse histopathology (Schofield et al., 2010), and the Mouse Tumor Biology (MTB) database (Bult et al., 2015) for images of neoplastic and hyperplastic lesions. Both are community resources – copyright remains with submitters – and both have been running for more than 15 years. Pathbase uses standard anatomical and pathological ontologies for metadata. There are also web services provided for computational access and, recently, the database has been moved onto the OMERO open source platform. Pathbase and the MTB database are both heavily accessed and open to user submission, but the number of images uploaded directly from the community as opposed to from targeted studies remains disappointingly low. An ongoing challenge for both databases is lack of external funding and, considering various sustainability models, it is clear that it would be difficult to maintain a long-term community role without external funding input. There are currently no established large-scale databases of histopathology, apart from MTB and Pathbase, that allow data uploading for free, allow users to retain rights over images and provide free access to all users. More recently, The Company of Biologists (publisher of Disease Models & Mechanisms) and other publishers have partnered with Dryad, a curated resource for the deposition of different types of data, to facilitate data sharing for all publication-associated data. Although this could help in addressing the wider issue of irreproducibility, as yet Dryad cannot accept whole-slide histopathology images and so the problem of making these data generally accessible through public, as opposed to local lab, resources remains highly important.

The ‘image problem’ thus remains an issue for the sharing of histopathology data and, given the scale of the problem, a substantial and collaborative commitment from funding agencies and publishers is needed in order to ensure that data are accessible so that the accuracy, accountability and reproducibility of results can be verified.

## Concluding remarks

In the first instance, reproducibility depends on informed expert review, which, like replication, depends on the disclosure and public sharing of primary data and comprehensive protocols. Qualified and experienced histopathologists are needed as part of the review process in cases where histopathology data is the main evidence for phenotypes. The absence of a platform for sharing such images, with appropriate discoverability, remains a problem that journals and funding agencies alike need to address in terms of funding support and increased awareness.

The impacts of strain, sex, age and allele, together with environmental factors, such as housing, diet, health status and microbiology, are highly significant and can radically change the interpretation of histopathology data. There needs to be increased awareness of these factors within the community but, as importantly, these parameters need to be captured and shared with publications. The ARRIVE guidelines cover all of the parameters identified here in detail and yet there is still a problem in persuading authors to provide this information and for journals to enforce their own policies. Inclusion of ARRIVE parameters in Laboratory Information Management System (LIMS) software might encourage reporting, as suggested by Karp et al. (2015), but an interesting possibility is for institutional animal facilities to publish overall husbandry conditions on their websites, or make an updated document available to referees and readers in which at least some of the key parameters, such as the source of standard chow, water treatment, light/dark cycles, SPF status and definition might be obtained. This is currently done for the scientists at The Jackson Laboratory because many of their research colonies are the primary source for mutants that are distributed to the community. The production of standard animal facility husbandry and disease status reports, put together by managers and supervising veterinarians, might address many of these issues efficiently. This would leave investigators only with the need to flag deviations from standard facility conditions in their experiments.

The bottom line, however, is that it is only with a firm commitment to disclosure and sharing by investigators, journals and funding agencies, and a recognition by the latter that, in many cases, ensuring reproducibility has a financial cost, that we will see better value for money from investment in model organism development and, in turn, a more robust translational pipeline.

## Footnotes

1 http://grants.nih.gov/reproducibility/index.htm, http://washingtonupdate.faseb.org/faseb-hosts-discussion-on-rigor-and-reproducibility-of-research-using-mouse-models/.

2 The Biosharing website, https://biosharing.org/, contains policy summaries for major funding agencies.
